# Sex-specific socioeconomic inequalities in trajectories of anthropometry, blood pressure, and blood-based biomarkers from birth to 18 years: a prospective cohort study

**DOI:** 10.1093/eurpub/ckaf022

**Published:** 2025-03-10

**Authors:** Kate N O’Neill, Minhal Ahmed, Linda M O’Keeffe

**Affiliations:** School of Public Health, University College Cork, Cork, Ireland; Harvard Medical School, Harvard University, Boston, MA, United States; School of Public Health, University College Cork, Cork, Ireland; MRC Integrative Epidemiology Unit at the University of Bristol, Bristol, United Kingdom; Population Health Sciences, Bristol Medical School, University of Bristol, Bristol, United Kingdom

## Abstract

Evidence on when socioeconomic inequalities in conventional cardiometabolic risk factors emerge and how these change over time is sparse but important in identifying pathways to socioeconomic inequalities in cardiovascular disease (CVD). We examine socioeconomic inequalities in cardiometabolic risk factors trajectories across childhood and adolescence. Data were from the Avon Longitudinal Study of Parents and Children (ALSPAC), born in 1991/1992. Socioeconomic position (SEP) was measured using maternal education from questionnaires at 32-weeks’ gestation. Cardiometabolic risk factors measured from birth/mid-childhood to 18 years (y) included fat and lean mass (9–18 y), systolic and diastolic blood pressure (SBP, DBP), pulse rate and glucose (7–18 y), high-density lipoprotein cholesterol (HDL-c), non-HDL-c and triglycerides (birth–18y). Associations were examined using linear spline multilevel models. Among 6517–8952 participants with 11 948–42 607 repeated measures, socioeconomic inequalities in fat mass were evident at age 9 y and persisted throughout adolescence. By 18 y, fat mass was 12.32% [95% confidence interval (CI): 6.96, 17.68] lower among females and 7.94% (95% CI: 1.91, 13.97) lower among males with the highest SEP compared to the lowest. Socioeconomic inequalities in SBP and DBP were evident at 7 y, narrowed in early adolescence and re-emerged between 16 and 18 y, particularly among females. Socioeconomic inequalities in lipids emerged, among females only, between birth and 9 y in non-HDL-c, 7 and 18 y in HDL-c, and 9 and 18 y in triglycerides while inequalities in glucose emerged among males only between 15 and 18 y. Prevention targeting the early life course may be beneficial for reducing socioeconomic inequalities in CVD especially among females who have greater inequalities in cardiometabolic risk factors than males at the end of adolescence.

## Introduction

Cardiovascular disease (CVD) is the leading cause of death globally [1]. CVD risk develops early in the life course [2, 3] and tracks from childhood through to adulthood [4, 5]. Socioeconomic inequalities in CVD are well established in later life [6, 7], little is known about when socioeconomic inequalities in key CVD risk factors emerge and how they change over time [8].

Despite decades of research their prevention remains a global health challenge [9] and continues to be prioritized in national and international health strategies [7]. Efforts to prevent or reduce socioeconomic inequalities in CVD have been curbed by a limited understanding of the pathways that likely drive such inequalities across the life course [8]. For instance, numerous studies have examined socioeconomic inequalities in adiposity in early life [10–15], but these have often only had measures available at a single time point or have studied trajectories of adiposity from early childhood but have not included risk factors such as blood pressure and lipids to examine the concomitant change together to better understand aetiology. For example, an analysis of the Avon Longitudinal Study of Parents and Children (ALSPAC) birth cohort study explored trajectories of ponderal index from birth to 2 years (y) and body mass index from 2 to 10 y demonstrating that socioeconomic inequalities in adiposity emerged around the age of 4 y and continued to widen with increasing age up to 10 y [14]. A recent analysis of the Millenium Cohort Study examined trajectories of fat mass from age 7 to 17 y and found socioeconomic inequalities were evident at age 7 y and continued to widen with age up to 17 y [16]. A further analysis of the ALSPAC cohort explored socioeconomic inequalities in trajectories of fat mass, blood pressure, and lipids and found inequalities in fat mass among females only and in cholesterol and blood pressure in both sexes. However, measurements were only available from age 9 to 15 y and it is not clear whether these patterns track into early adulthood [13].

Understanding the socioeconomic patterning of cardiometabolic risk factors across the life course can provide important insights into when socioeconomic inequalities emerge and how they change over time [8], thus identifying opportunities for the prevention of socioeconomic inequalities in CVD. For instance, differences that are evident in early childhood may implicate familial factors related to early life adversity or early childhood nutrition while differences emerging around puberty could suggest a role for health behaviours such as smoking and physical activity. We use a contemporary prospective birth cohort study to examine socioeconomic inequalities in trajectories of cardiometabolic risk factors across childhood and adolescence, from birth to 18 y. Risk factors include height-adjusted fat and lean mass (9–18 y), systolic blood pressure (SBP), diastolic blood pressure (DBP), pulse rate and glucose (7–18 y), high-density lipoprotein cholesterol (HDL-c), non-high-density lipoprotein cholesterol (non-HDL-c), and triglycerides (birth–18 y).

## Methods

### Study participants

Data were from first-generation children of ALSPAC, a population-based prospective birth cohort study in southwest England [17, 18]. Pregnant women resident in one of three Bristol-based health districts with an expected delivery date between 1 April 1991 and 31 December 1992 were invited to participate. ALSPAC initially enrolled a cohort of 14 451 pregnancies, from which 14 062 live births occurred, and 13 988 children were alive at age 1 y. When the oldest children were approximately 7 y, an attempt was made to bolster the sample with eligible cases who had not joined the study originally. Therefore, the total sample size for analyses using data collected after the age of 7 y is 14 901 children. Follow-up has included parent- and child-completed questionnaires, research clinic attendance, and links to routine data. Ethical approval for the study was obtained from ALSPAC Ethics and Law Committee and the Local Research Ethics Committees. Informed consent for the use of data collected via questionnaires and clinics was obtained from participants following the recommendations of the ALSPAC Ethics and Law Committee at the time. The study website contains details of all the data that is available through a fully searchable data dictionary and variable search tool—http://www.bristol.ac.uk/alspac/researchers/our-data/.

### Socioeconomic position

Socioeconomic position (SEP) is a construct encompassing multiple dimensions of social inequality. SEP is usually defined by indicators including educational attainment, occupational class, and/or material circumstances [19]. Maternal educational attainment was used as an indicator of SEP in this analysis as it is the most complete measure available in ALSPAC and is the most frequently reported indicator of childhood SEP [14, 15, 20]. At 32-weeks’ gestation, mothers were asked to report their highest educational attainment based on UK standards at the time which was categorized as ‘less than O-level’ (ordinary level; exams taken usually at age 15 y or 16 y at the completion of legally required school attendance, equivalent to the present UK General Certificate of Secondary Education; assumed to reflect the lowest SEP), ‘O-level’, ‘A-level’ (advanced level; exams taken usually at age 18 y), or ‘university degree’ (undergraduate or postgraduate; assumed to reflect the highest SEP).

### Cardiometabolic risk factor measurement

#### Fat and lean mass

Whole body less head, and central fat and lean mass were derived from whole body dual energy X-ray absorptiometry (DXA) scans assessed five times (ages 9, 11, 13, 15, and 18 y) using a Lunar prodigy narrow fan beam densitometer.

#### SBP, DBP, and pulse

At each clinic (ages 7, 9, 10, 11, 12, 15, and 18 y), SBP, DBP, and pulse rate were measured at least twice with the child sitting and at rest with the arm supported, using a cuff size appropriate for the child’s upper arm circumference and a validated blood pressure monitor. The mean of the two final measures was used.

#### Blood based biomarkers

Non-fasting glucose was measured at age 7 y as part of metabolic trait profiling, using nuclear magnetic resonance (NMR) spectroscopy. In a random 10% of the cohort at age 9 y, fasting glucose was also available [17]. Fasting glucose was available from research clinics held when participants were aged 15 and 18 y. HDL-c, total cholesterol, and triglycerides were measured in cord blood at birth and from venous blood subsequently. Samples were non-fasted at 7 and 9 y; fasting measures were available from clinics at 15 and 18 y. Non-HDL-c was calculated by subtracting HDL-c from total cholesterol at each measurement occasion.

### Statistical analysis

We used multilevel models to examine the association between SEP and change in each risk factor across childhood and adolescence. Sex-specific trajectories of all outcomes have been modelled previously using multilevel models and are described elsewhere in detail [2, 21–23]. We included all participants with data on sex, SEP, and at least one measurement of the risk factor throughout the study period in each multilevel model, under a missing-at-random (MAR) assumption [24, 25]. Model fit statistics for each risk factor trajectory are shown in Supplementary Tables S1–S7. Using a directed acyclic graph (DAG) to illustrate our causal assumptions related to this research question, we did not identify any potential confounders (as distinct from mediators and colliders) and therefore we do not include any additional variables in our models other than SEP, each risk factor, and sex [26].

To explore the sex-specific associations between SEP and cardiometabolic risk factors, maternal education of less than O-level was selected as the reference category. An interaction between each of the other maternal education categories and the intercept and each linear spline period was included in all models to estimate the differences in intercepts and slopes between each maternal education category and the reference group. All models included interactions between sex and the intercept and linear splines to allow trajectories to differ in females and males and between sex, SEP, and the intercept and linear splines to allow associations with SEP to differ in females and males.

In all models, age (in years) was centred at the first available measure. Fat mass and lean mass were adjusted for height using the time- and sex-varying power of height that best resulted in a height-invariant measure, as described previously [21]. Values of cardiometabolic risk factors that had a skewed distribution (fat mass, triglycerides) were (natural) log transformed prior to analyses. Differences and confidence intervals were calculated on the log-scale and were back-transformed therefore interpreted as the ratio of geometric means. Graphs displayed for these risk factors are in original units and derived by back transforming from the natural log-scale. For ease of interpretation, trajectories for the highest SEP (degree level maternal education) and the lowest SEP (less than O-level maternal education) are displayed in graphs while full results are presented in Supplementary Material S1. All trajectories were modelled in MLwiN version 3.04, called from Stata version 17 using the runmlwin command [27].

## Results

The number of participants included in analyses ranged from 8952 participants (19 938 measures) for triglycerides to 6517 participants (11 948 measures) for glucose (Supplementary Table S8 and Fig. S1). Characteristics of participants are detailed in Table 1. Maternal education was similar in females and males, with approximately a quarter of mothers having less than O-level educational attainment.

**Table 1.** Characteristics of ALSPAC participants included in the analysis, by sex

	Females *N* = 4377^a^	Males *N* = 4575^a^
	*n* (%)	*n* (%)
Maternal marital status		
Never married	675 (15.8)	673 (15.1)
Separated/divorced/widowed	210 (4.9)	243 (5.4)
1st marriage	3129 (73.1)	3239 (72.6)
Marriage 2 or 3	268 (6.3)	308 (6.9)
Household social class^b^		
Professional	596 (14.4)	641 (15.0)
Managerial & technical	1781 (43.0)	1851 (43.3)
Non-manual	1040 (25.1)	1070 (25.0)
Manual	501 (12.1)	507 (11.9)
Part skilled & unskilled	223 (5.4)	207 (4.8)
Maternal education		
Less than O-level	1120 (25.6)	1189 (26.0)
O-level	1529 (34.9)	1610 (35.2)
A-level	1072 (24.5)	1126 (24.6)
Degree or above	656 (15.0)	650 (14.2)
Mother’s partner’s highest educational qualification		
Less than O-level	1343 (31.7)	1332 (30.2)
O-level	905 (21.4)	961 (21.8)
A-level	1150 (27.2)	1199 (27.2)
Degree or above	834 (19.7)	912 (20.7)
Maternal smoking during pregnancy		
No	3438 (80.0)	3527 (78.6)
Yes	858 (20.0)	961 (21.4)
Parity		
0	1902 (44.3)	1957 (43.4)
1	1549 (36.1)	1699 (35.4)
2	844 (19.7)	955 (21.2)
Breastfeeding		
Exclusive	1313 (33.7)	1227 (30.2)
Non-exclusive	1818 (46.6)	2048 (50.3)
Never	769 (19.7)	793 (19.5)
	Mean (SD)	Mean (SD)
Gestational age (weeks)	40 (1.7)	39 (1.9)
Birth weight (g)	3372 (502.5)	3483 (559.0)
Maternal pre-pregnancy BMI (kg/m^2^)	23 (3.5)	23 (3.4)
Maternal age at delivery (years)	29 (4.7)	29 (4.8)

a Denominators in this table do not sum exactly to *N* participants included in models due to some missing covariate data which was not required for inclusion in our analyses. Number of participants available for analyses of triglycerides (*n* = 8952) used as the denominator in this table given the varying sample sizes included in analyses.

b Household social class was measured as the highest of the mother’s or her partner’s occupational social class using data on job title and details of occupation. Social class was derived using the standard occupational classification (SOC) codes developed by the United Kingdom Office of Population Census and Surveys and classified as I professional, II managerial and technical, IIINM non-manual, IIIM manual, and IV&V part skilled occupations and unskilled occupations.

### Fat and lean mass

**Figure 1.**
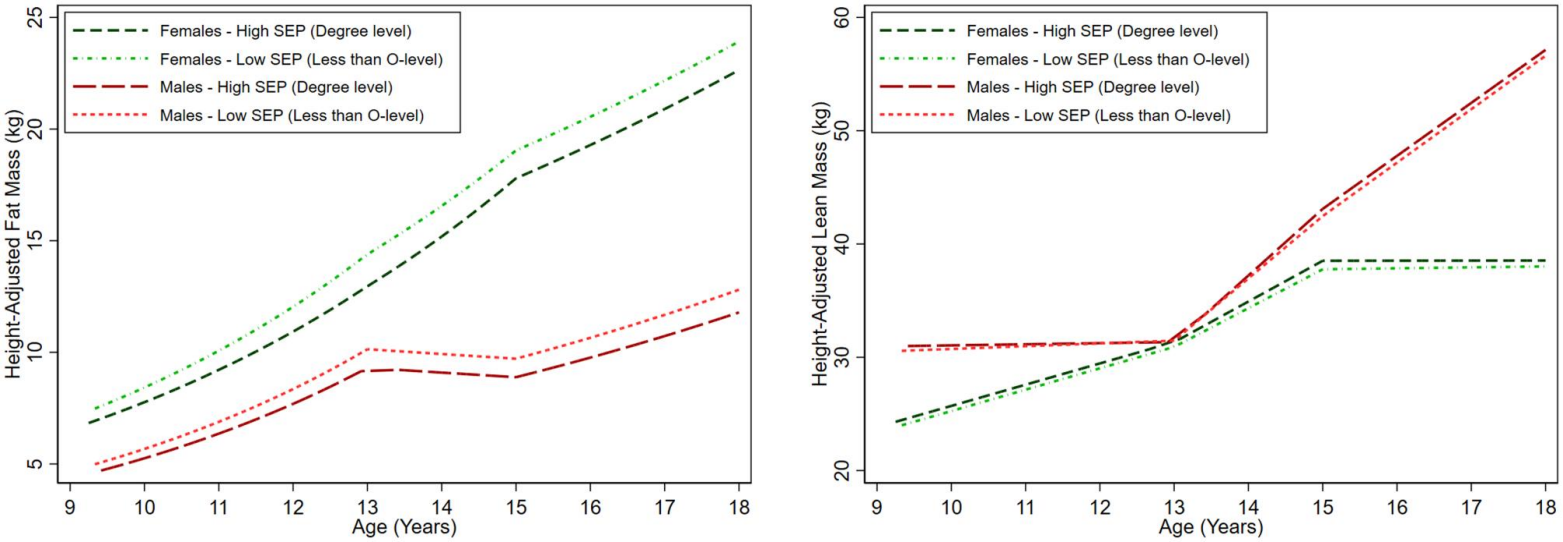
Mean trajectories of fat mass and lean mass in females and males, by maternal education, estimated from multilevel models. Detailed results for the mean trajectories among those with less than O-level maternal education and mean differences among those with O-level, A-level, and degree level maternal education along with 95% confidence intervals are provided in Supplementary Tables S10 and S11. kg, kilograms; SEP, socioeconomic position.

Among females, there was evidence of a socioeconomic gradient in fat mass across all maternal education categories at age 9 y with some evidence of inequalities widening over time (Fig. 1, Supplementary Tables S9 and S10). For instance, at 9 y fat mass was 3.0% [95% confidence interval (CI): −1.60, 7.60], 4.99% (95% CI: 0.20, 9.78), and 10.61% (95% CI: 5.38, 15.75) lower among females with O-level, A-level, and degree level maternal education, compared with the lowest maternal education (less than O-level), respectively, with associations strengthening to 4.68% (95% CI: −0.21, 9.57), 8.43% (95% CI: 3.44, 13.42), and 12.32% (95% CI: 6.96, 17.68) by 18 y. Among males, lower fat mass was only evident in those with the highest maternal education (degree level) compared with the lowest (less than O-level) at age 9 y and associations were similar at 18 y (Fig. 1, Supplementary Tables S9 and S10). For instance, degree level maternal education was associated with 7.08% (95% CI: −1.66, 12.50) and 7.94% (95% CI: 1.91, 13.97) lower fat mass at age 9 and 18 y, respectively. Among females, higher lean mass was evident among those with highest maternal education compared to the lowest at 18 y only [difference: 0.51 kg (95% CI: −0.09, 1.11)] (Fig. 1, Supplementary Tables S9 and S10). Higher lean mass at 9 y was evident among males with higher maternal education, and this persisted at 18 y.

### SBP, DBP, and pulse

**Figure 2.**
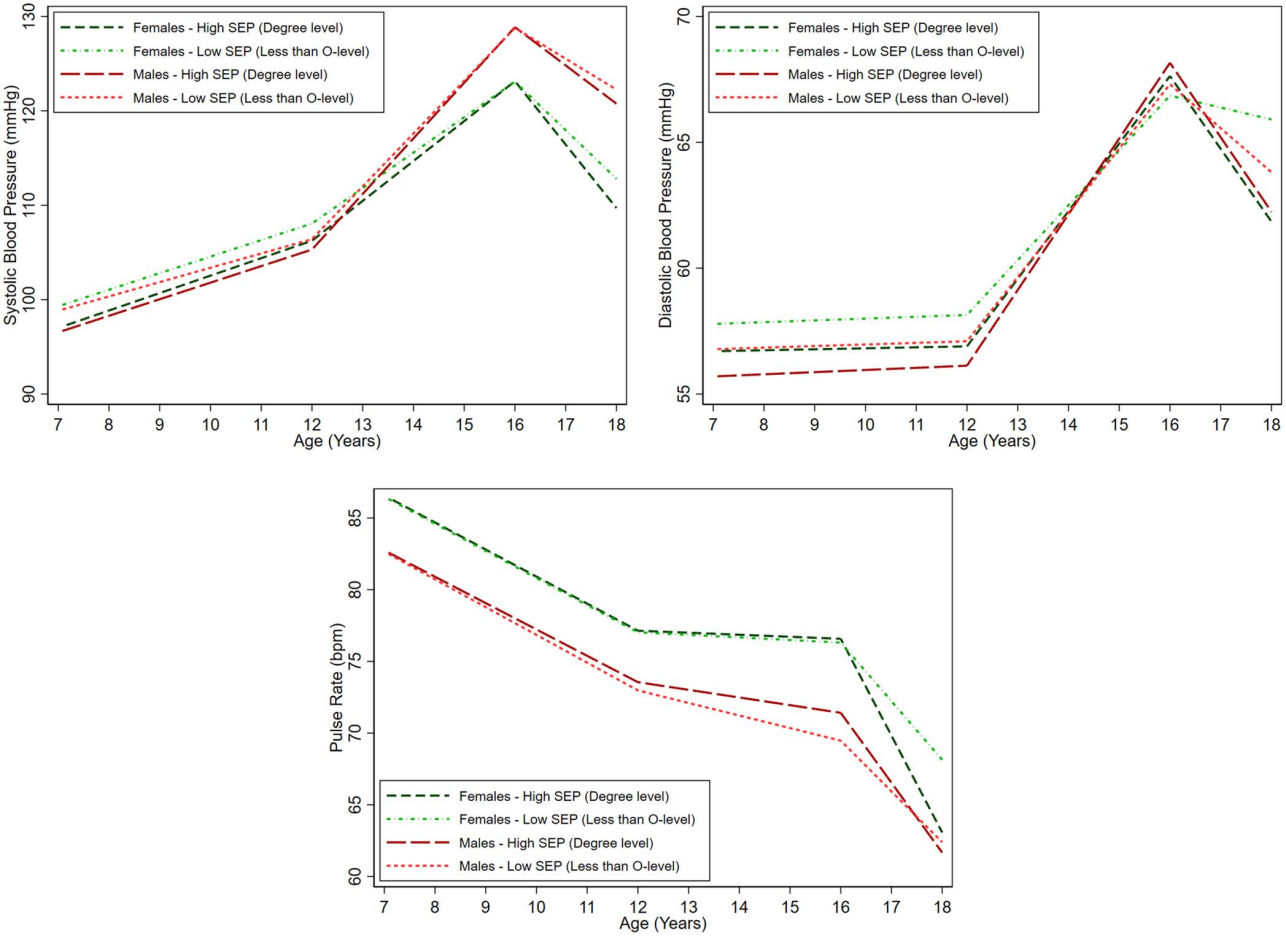
Mean trajectories of SBP, DBP, and pulse in females and males, by maternal education, estimated from multilevel models. Detailed results for the mean trajectories among those with less than O-level maternal education and mean differences among those with O-level, A-level, and degree level maternal education along with 95% confidence intervals are provided in Supplementary Table S12. DBP, diastolic blood pressure; SBP, systolic blood pressure; mmHg, millimetres of mercury; bpm, beats per minute; SEP, socioeconomic position.

Among females, there was evidence of a socioeconomic gradient in SBP across all maternal education categories at age 7 y (Fig. 2, Supplementary Table S11). For instance, higher maternal education of O-level, A-level, and degree level was associated with 0.98 mmHg (95% CI: 0.16, 1.79), 1.71 mmHg (95% CI: 0.85, 2.57), and 2.32 mmHg (95% CI: 1.34–3.29) lower SBP, respectively, compared with the lowest maternal education (less than O-level). Faster increases in SBP were observed among those with the highest maternal education compared with the lowest from 12 to 16 y but from 16 to 18 y there was evidence of faster decreases in SBP among those with O-level, A-level, and degree level compared with the lowest maternal education giving rise to widening inequalities by 18 y. At 18 y, O-level, A-level, and degree level maternal education were associated with 1.30 mmHg (95% CI: 0.31, 2.29), 2.74 mmHg (95% CI: 1.69, 3.80), and 3.04 mmHg (95% CI: 1.88–4.21) lower SBP, respectively among females. Associations were broadly similar among males at age 7 y with evidence of a socioeconomic gradient in SBP across all maternal education categories. Faster increases in SBP were observed among those with A-level and degree level maternal education compared with the lowest (less than O-level) from 7 to 12 y. Faster decreases in SBP were observed among those with the highest maternal education (degree level) only from 16 to 18 y such that by 18 y, associations had weakened across maternal education categories and lower SBP was only observed in those with degree level maternal education compared with less than O-level [difference: −1.50 mmHg (95% CI: −2.80, −0.19)]. Similar patterns of associations were observed for DBP (Fig. 2, Supplementary Table S11).

There was no evidence of associations with pulse at age 7 y among females (Fig. 2, Supplementary Table S11). Faster decreases were observed among females with degree level and A-level maternal education between ages 16 and 18 y compared with the lowest maternal education (less than O-level) resulting in a lower pulse rate of 2.74 bpm (95% CI: 1.45–4.03) and 2.74 bpm (95% CI: 1.58, 3.91), respectively, by age 18 y. There was no evidence of associations with pulse from 7 to 18 y among males (Fig. 2, Supplementary Table S11).

### Blood based biomarkers

**Figure 3.**
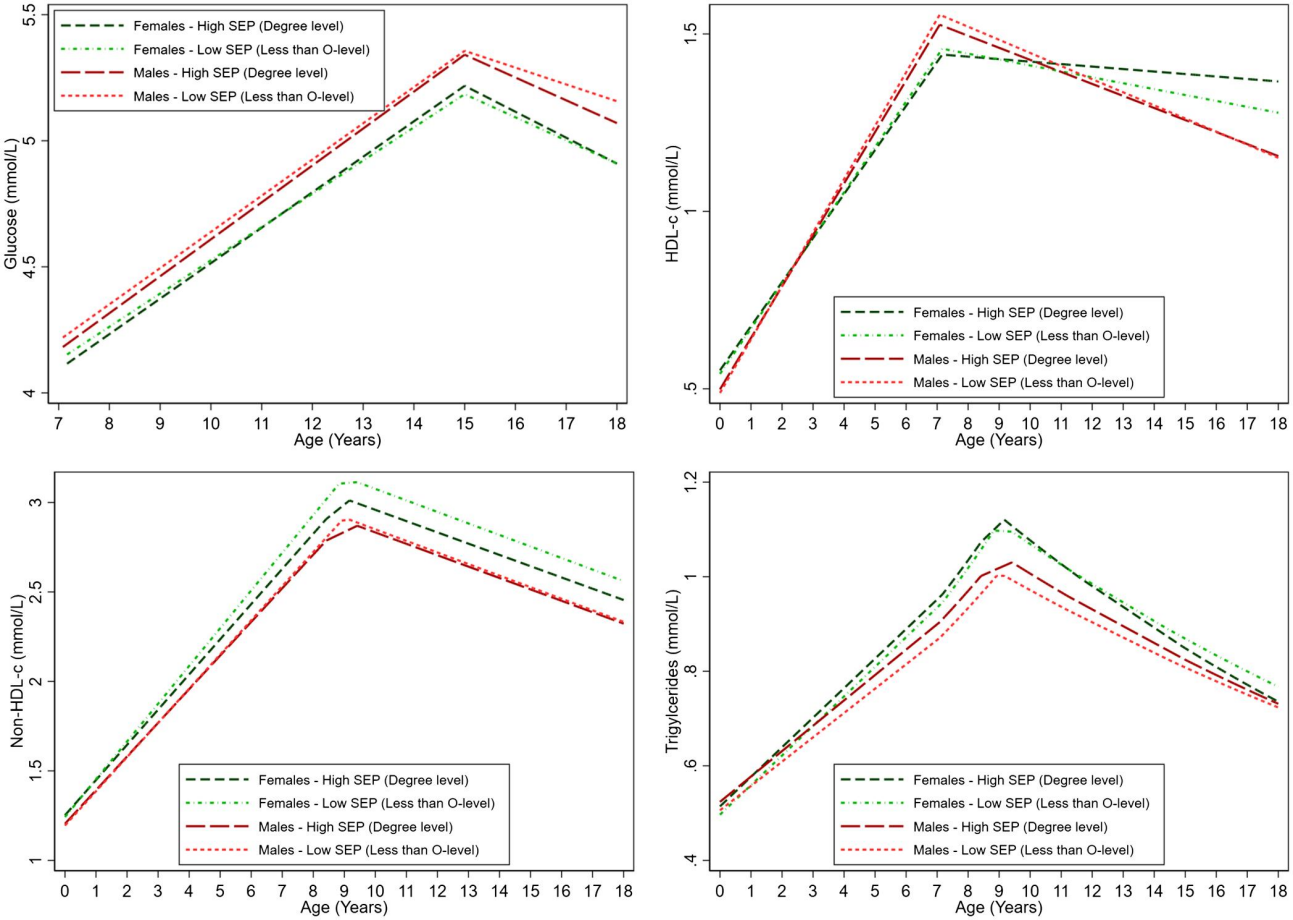
Mean trajectories of glucose, HDL-c, non-HDL-c, and triglycerides in females and males, by maternal education, estimated from multilevel models. Detailed results for the mean trajectories among those with less than O-level maternal education and mean differences among those with O-level, A-level, and degree level maternal education along with 95% confidence intervals are provided in Supplementary Tables S13–S15. HDL-c, high-density lipoprotein cholesterol; non-HDL-c, non-high-density lipoprotein cholesterol; mmol/l, millimole per litre; SEP, socioeconomic position.

There was little evidence of associations of maternal education and glucose trajectories except for 0.09 mmol/l (95% CI: 0.02–0.16) lower glucose among males with the highest maternal education (degree level) at 18 y compared with the lowest (less than O-level) (Fig. 3, Supplementary Table S12). This was driven by faster decreases among those with degree level maternal education between ages 15 and 18 y.

Between birth and 9 y, slower increases in non-HDL-c were observed among females with higher maternal education such that by age 18 y, non-HDL-c was 0.05 mmol/l (95% CI: −0.02, 0.12), 0.09 mmol/l (95% CI: 0.01, 0.16), and 0.09 mmol/l (95% CI: 0.01, 0.18) lower among those with A-level, O-level, and degree level maternal education compared with the lowest (less than O-level), respectively (Fig. 3, Supplementary Table S13). Associations of maternal education and HDL-c emerged among females due to slower decreases among those with higher maternal education between 7 and 18 y such that by 18 y, O-level, A-level, and degree level was associated with 0.03 mmol/l (95% CI: 0.001, 0.06), 0.05 mmol/l (95% CI: 0.02, 0.08), and 0.11 mmol/l (95% CI: 0.07, 0.15) higher HDL-c compared with the lowest maternal education (less than O-level), respectively (Fig. 3, Supplementary Table S13). Maternal education was not associated with non-HDL-c and HDL-c trajectories from birth to 18 y among males. Associations of maternal education and triglycerides emerged between 9 and 18 y among females due to faster decreases among those with degree level maternal education compared with less than O-level (Fig. 3, Supplementary Table S14). By 18 y, the highest maternal education (degree level) was associated with 7.11% (95% CI: 2.35, 11.86) lower triglycerides compared with the lowest (less than O-level). There was little evidence of associations with triglycerides from birth to 18 y among males.

## Discussion

Socioeconomic inequalities in fat mass and blood pressure were evident in childhood and persisted throughout adolescence, with graded associations across all levels of maternal education among females but not males. Adolescence was an important period for the emergence of socioeconomic inequalities in pulse, HDL-c, and triglycerides among females only while inequalities in glucose emerged among males only.

Similar to previous findings in ALSPAC [14, 15], socioeconomic inequalities in fat mass were evident at age 9 y in both sexes and there was evidence of a SEP gradient in associations among females only whereby fat mass was lower across increasing categories of maternal education. In contrast, among males, socioeconomic inequalities in fat mass were evident only in those with degree level maternal education and there was little SEP gradient across all lower levels of maternal education. We show that this childhood pattern continues into adolescence and that there was some evidence of inequalities widening across all categories of maternal education among females by 18 y while remaining similar over time among males. Our findings are consistent with a recent systematic review, including 50 studies mostly from high-income countries, that reported socioeconomic inequalities in fat mass in childhood that were wider among females compared with males [10]. Analyses of the Millennium Cohort Study in the UK also found greater evidence of inequalities in fat mass in females early in childhood, but in contrast to our findings, inequalities widened at a faster rate in males so that by late adolescence, inequalities in females and males were similar [16]. However, area-level deprivation was used as the indicator of SEP in Millennium Cohort Study which reportedly captures elements of the environment beyond family SEP in adolescence, but not in early childhood. Taken together, these findings may suggest that different dimensions of SEP influence risk in females and males across the early life course. SEP throughout the life course has previously been shown to influence CVD risk differently in adult females and males [28].

Similar to a previous ALSPAC analysis that explored associations of maternal education and blood pressure at age 10 y [15], we found socioeconomic inequalities in blood pressure in childhood. A further analysis of approximately 8500 children in ALSPAC demonstrated that inequalities in blood pressure narrowed with age from 7 to 15 y [13]. However, while our findings also suggest that inequalities in blood pressure narrowed throughout childhood and early adolescence, we found faster rates of decreases in SBP and DBP among those with higher maternal education between ages 16 and 18 y that resulted in a re-emergence of socioeconomic inequalities in SBP and DBP by 18 y, particularly among females. Similar to patterns observed in fat mass, associations were graded across levels of maternal education among females but not males. It has been posited that early influences of SEP on child health may be reduced in adolescence due to the influence of school environment, peer support, and youth culture, in what has been termed an equalization of health during youth, but that inequalities re-emerge thereafter [29]. However, further work is required to determine the underlying mechanisms and whether changes over time are indeed explained by a theory of equalization or by biological phenomenon such as the transient effect of puberty on blood pressure [30], the timing of which may differ by SEP [31]. Our findings also identify sex-specific patterns in socioeconomic inequalities in lipids and glucose. Previous research in ALSPAC reported little evidence of socioeconomic inequalities in cholesterol and triglycerides at age 9 y in females and males [15]. However, we found that socioeconomic inequalities emerged in HDL-c and triglycerides in adolescence, and in non-HDL-c in childhood among females only. We also found evidence of socioeconomic inequalities in glucose that emerged in adolescence among males. With limited evidence on socioeconomic inequalities in glucose in early life [32], this finding requires further exploration in other cohorts.

Taken together, our findings provide important insights into the potential aetiological pathways for socioeconomic inequalities in CVD. The different patterns of inequalities in fat mass and blood pressure in adolescence indicate that adiposity may not fully explain the observed associations of SEP and blood pressure. Inequalities in fat mass persisted among males and somewhat strengthened among females between 9 and 18 y while inequalities in SBP and DBP narrowed between the ages over this time only to re-emerge between 16 and 18 y. In contrast, the emergence of socioeconomic inequalities in lipids among females in adolescence particularly may indicate a role for adiposity given the suggestive evidence of widening inequalities in fat mass among females over this period. Although there is limited understanding of how SEP influences CVD risk across the life course [33, 34], a recent Mendelian randomization study found that three established risk factors including adiposity and systolic blood pressure may explain up to half of the effect of SEP on CVD [35]. Our findings of socioeconomic inequalities in these two risk factors in childhood and adolescence therefore indicate that early life may be an important period for the prevention of a significant proportion of socioeconomic disparities in CVD. However, future work exploiting the maturation of contemporary birth cohort studies is required to determine whether such inequalities in early life track into adulthood. Alongside this, advancements in mediation analysis methodologies provide opportunities to formally examine whether and how socioeconomic inequalities in CVD risk factors across the life course influence CVD outcomes and therefore allow for the identification of potential intervention targets at appropriate timepoints across the life course [8, 36, 37].

### Strengths and limitations

Strengths of our study include the availability of repeated measures of cardiometabolic risk factors from birth/early life to 18 y and the use of multilevel models allowing for clustering of repeated measures within individuals and correlation between measures over time. Multilevel models also allow for the inclusion of all participants with at least one measure of a cardiometabolic risk factor throughout the follow-up period, thereby minimizing potential for selection bias and increasing our sample size. The study also has some limitations. The possibility of selection bias remains given that participants included in our analysis were more likely to have higher maternal education levels compared with those excluded due to missing data (Supplementary Table S15). Loss to follow-up is another potential limitation of our study. However, we have included all participants with at least one measure of each risk factor to minimize any potential selection bias. ALSPAC is a contemporary prospective cohort study, however SEP data refer to childhood SEP in the early 1990s. The meaning of SEP indicators such as education change over time and as such likely cohort effects need to be considered in the interpretation of findings.

## Conclusion

Socioeconomic inequalities in fat mass and blood pressure emerge in childhood for both sexes and in adolescence for lipids and pulse rate among females and glucose among males, with some evidence of stronger graded associations among females compared with males. Prevention targeting the early life course may be beneficial for reducing socioeconomic inequalities in CVD especially among females.

## Supplementary Material

ckaf022_Supplementary_Data

## Data Availability

While the ALSPAC data used in these analyses are de-identified, there are legal restrictions on sharing these data imposed by the custodians of the data, The University of Bristol. Further information can be found here: http://www.bristol.ac.uk/media-library/sites/alspac/documents/researchers/data-access/ALSPAC_Access_Policy.pdf. All data enquiries can be sent to: ALSPAC-exec@bristol.ac.uk; Tel:+44(0)117 331 0167. Key points Socioeconomic inequalities in fat mass and blood pressure were evident in childhood and persisted throughout adolescence, particularly among females. Adolescence was an important period for the emergence of socioeconomic inequalities for lipids and pulse rate among females and glucose among males. Prevention targeting the early life course may be beneficial for reducing socioeconomic inequalities in CVD especially among females.
