# Supplementary material for: Sex-specific socioeconomic inequalities in trajectories of anthropometry, blood pressure, and blood-based biomarkers from birth to 18 years: a prospective cohort study
Source: Eur J Public Health. 2025 Mar 10;35(2):249–55. doi: 10.1093/eurpub/ckaf022 (PMC11967908; doi:10.1093/eurpub/ckaf022)
Supplement: ckaf022_Supplementary_Data [file ckaf022_supplementary_data.pdf]

Sex-specific socioeconomic inequalities in trajectories of anthropometry, blood pressure and blood-based biomarkers from birth to 18 years: a prospective cohort study

Supplementary Material

Supplementary Material Contents

Table S1: Model details for log fat mass trajectories
Table S2: Model details for lean mass trajectories
Table S3: Model details for SBP, DBP and pulse rate trajectories
Table S4: Model details for glucose trajectories
Table S5: Model details for HDL-c trajectories
Table S6: Model details for non-HDL-c trajectories
Table S7: Model details for log triglyceride trajectories
Figure S1: Flow diagram of study
Table S8: Number of participants with cardiometabolic risk factor measures at each time point
Table S9: Mean trajectories of log fat mass and mean differences by maternal education, estimated from multilevel models
Table S10: Mean trajectories of lean mass and mean differences by maternal education, estimated from multilevel models
Table S11: Mean trajectories of SBP, DBP and pulse and mean differences by maternal education, estimated from multilevel models
Table S12: Mean trajectories of glucose and mean differences by maternal education, estimated from multilevel models
Table S13: Mean trajectories of HDL-c and non-HDL-c and mean differences by maternal education, estimated from multilevel models
Table S14: Mean trajectories of log triglycerides and mean differences by maternal education, estimated from multilevel models
Table S15: Characteristics at birth of the mothers of children included in models compared with those excluded due to missing exposure or outcome data

Table S1: Model details for log fat mass trajectories

	Number of contributing individuals		Assessment of model fit			
	Total observations	Individuals with 1 measure	Mean observed, ln(kg) (SD)[a]	Mean predicted, ln(kg) (SD)[aa]	Mean difference (observed – predicted), ln(kg)[a]	95% level of agreement between observed and predicted, ln(kg)[a]
Overall	27609	7887				
9 years	6602	6602	1.97 (0.57)	1.97 (0.54)	-0.002	-0.18 to 0.17
9-13 years	12972	7426	2.13 (0.59)	2.13 (0.56)	-0.0004	-0.20 to 0.20
13-15 years	5573	5531	2.44 (0.59)	2.44 (0.56)	0.002	-0.25 to 0.25
15-18 years	9064	5607	2.64 (0.61)	2.64 (0.58)	-0.001	-0.18 to 0.18

SD, standard deviation; ln(kg), natural log of kilograms

[a]Fat mass is presented in the natural log and values represent the mean predicted natural log of fat mass.

Table S2: Model details for lean mass trajectories

	Number of contributing individuals		Assessment of model fit			
	**Total observations**	**Individuals with 1 measure**	**Mean observed, kg (SD)**	**Mean predicted, kg (SD)**	**Mean difference (observed – predicted), kg**	**95% level of agreement between observed and predicted, kg**
Overall	27676	7900				
9 years	6619	6619	24.55 (3.20)	24.49 (2.72)	0.06	-3.30 to 3.43
9-13 years	13001	7439	27.10 (4.62)	27.12 (4.30)	-0.03	-3.15 to 3.09
13-15 years	5583	5541	37.97 (6.43)	37.90 (6.48)	0.07	-3.62 to 3.75
15-18 years	9092	5621	44.25 (9.26)	44.25 (8.85)	-0.003	-2.19 to 2.18

SD, standard deviation; kg, kilograms

Table S3: Model details for SBP, DBP and pulse rate trajectories

	Number of contributing individuals		Assessment of model fit			
	Total observations	Individuals with 1 measure	Mean observed SBP, DBP or pulse rate (SD) [a]	Mean predicted SBP, DBP or pulse rate (SD) [a]	Mean difference (observed–predicted) [a]	95% level of agreement between observed and predicted [a]
**SBP**						
Overall	42368	8801				
7 years	7320	7320	98.83 (9.13)	98.71 (5.38)	0.12	-10.63 to 10.88
7-12 years	26674	8499	102.60 (9.53)	102.77 (6.18)	-0.17	-11.81 to 11.48
12-16 years	11153	6573	115.75 (11.67)	115.30 (8.74)	0.45	-11.31 to 12.21
16-18 years	4541	4396	116.89 (10.14)	117.21 (7.34)	-0.32	-12.59 to 11.96
**DBP**						
Overall	42370	8801				
7 years	7318	7318	56.45 (6.59)	56.68 (3.40)	-0.23	-8.96 to 8.50
7-12 years	26688	8498	58.10 (6.97)	56.96 (3.40)	1.15	-9.12 to 11.41
12-16 years	11147	6573	61.11 (9.58)	61.99 (5.11)	-0.87	-12.45 to 10.71
16-18 years	4535	4388	64.25 (6.11)	64.57 (3.64)	-0.33	-13.09 to 12.44
**Pulse**						
Overall	42607	8810				
7 years	7315	7315	83.27 (10.71)	83.26 (6.13)	0.01	-13.18 to 13.21
7-12 years	26905	8510	77.63 (11.60)	78.98 (6.99)	-1.35	-16.07 to 13.37
12-16 years	11163	6577	74.04 (11.25)	74.13 (6.91)	-0.09	-14.04 to 13.87
16-18 years	4539	4393	65.81 (10.16)	66.06 (6.26)	-0.25	-14.68 to 14.18

DBP, diastolic blood pressure; SBP, systolic blood pressure; SD, standard deviation

[a]units are presented in mmHg for SBP and DBP and bpm for pulse rate

Table S4: Model details for glucose trajectories

	Number of contributing individuals		Assessment of model fit			
	Total observations	Individuals with 1 measure	Mean observed, mmol/l (SD)	Mean predicted, mmol/l (SD)	Mean difference (observed – predicted), mmol/l	95% level of agreement between observed and predicted, mmol/l
Overall	11948	6517				
7 years	4965	4965	4.18 (0.50)	4.22 (0.23)	-0.04	-0.60 to 0.53
7-15 years	5822	5168	4.29 (0.55)	4.28 (0.26)	0.01	-0.59 to 0.62
15-18 years	6126	4123	5.12 (0.39)	5.13 (0.16)	-0.01	-0.65 to 0.63

SD, standard deviation; mmol/l, millimole per litre

Table S5: Model details for HDL-c trajectories

	Number of contributing individuals		Assessment of model fit			
	Total observations	Individuals with 1 measure	Mean observed, mmol/l (SD)	Mean predicted, mmol/l (SD)	Mean difference (observed – predicted), mmol/l	95% level of agreement between observed and predicted, mmol/l
Overall	19938	8928				
Birth	4183	4183	0.52 (0.23)	0.52 (0.10)	-0.000001	-0.26 to 0.26
0-7 years	4185	4185	1.06 (0.56)	1.05 (0.52)	0.01	-0.25 to 0.26
7-18 years	15753	7153	1.39 (0.32)	1.39 (0.24)	-0.00002	-0.25 to 0.25

HDL-c, high density lipoprotein cholesterol; SD, standard deviation; mmol/l, millimole per litre

Table S6: Model details for non-HDL-c trajectories

	**Number of contributing individuals**		**Assessment of model fit**			
	Total observations	Individuals with 1 measure	Mean observed, mmol/l (SD)	Mean predicted, mmol/l (SD)	Mean difference (observed – predicted), mmol/l	95% level of agreement between observed and predicted, mmol/l
Overall	19878	8919				
Birth	4128	4128	1.22 (0.53)	1.23 (0.18)	-0.01	-0.72 to 0.70
0-9 years	9045	7460	2.07 (0.98)	2.01 (0.77)	0.06	-0.63 to 0.74
9-18 years	10833	5977	2.63 (0.67)	2.68 (0.50)	-0.05	-0.66 to 0.56

Non-HDL-c, non-high density lipoprotein cholesterol; SD, standard deviation; mmol/l, millimole per litre

Table S7: Model details for log triglyceride trajectories

	Number of contributing individuals		Assessment of model fit			
	Total observations	Individuals with 1 measure	Mean observed, ln(trig), (SD)[a]	Mean predicted, ln(trig), (SD)[a]	Mean difference (observed – predicted), ln(trig)[a]	95% level of agreement between observed and predicted, ln(trig)[a]
Overall	19938	8952				
Birth	4267	4267	-0.68 (0.45)	-0.68 (0.21)	-0.003	-0.47 to 0.46
0-9 years	9160	7510	-0.35 (0.54)	-0.35 (0.37)	0.01	-0.52 to 0.53
9-18 years	10778	5961	-0.14 (0.41)	-0.14 (0.22)	-0.005	-0.54 to 0.53

ln(trig), natural log of triglyceride; SD, standard deviation

[a]Triglyceride is presented in the natural log and values represent the mean predicted natural log of triglyceride at each age shown.

Table S8: Number of participants with cardiometabolic risk factor measures at each time point

	Birth	Age 7	Age 9	Age 10	Age 11	Age 12	Age 13	Age 15	Age 18
Fat mass			6,602		6,364		5,539	4,734	4,370
Lean mass			6,619		6,376		5,549	4,750	4,382
SBP		7,320	6,920	6,556	6,405	6,069		4,879	4,219
DBP		7,318	6,927	6,562	6,410	6,066		4,874	4,213
Pulse rate		7,315	6,922	6,794	6,402	6,100		4,855	4,219
Glucose		4,965	824					3,181	2,978
HDL-c	4,183	4,919	4,657					3,192	2,987
Non-HDL-c	4,128	4,918	4,654					3,192	2,986
Triglycerides	4,267	4,894	4,629					3,180	2,968

HDL, high density lipoprotein cholesterol; DBP, diastolic blood pressure; SBP, systolic blood pressure.

Figure S1: Flow diagram of study

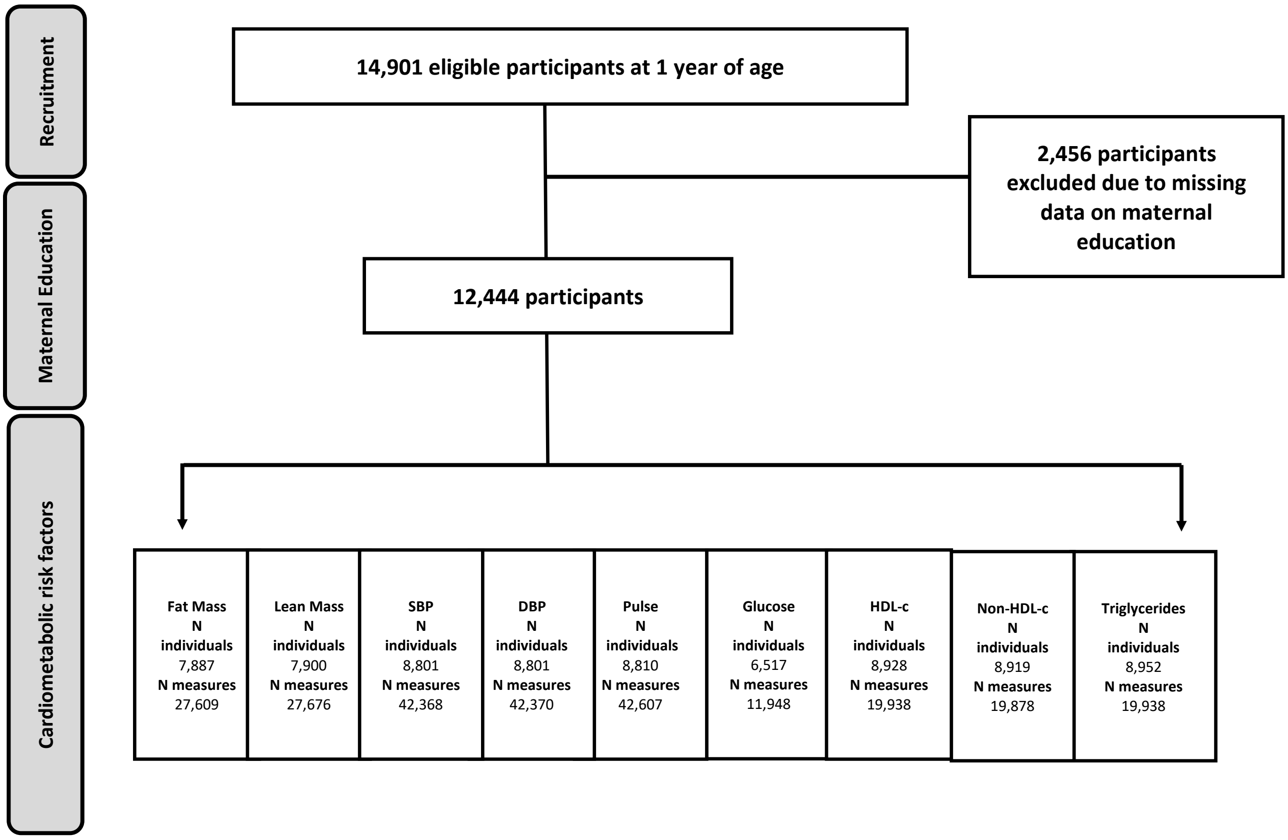

SBP, systolic blood pressure; DBP, diastolic blood pressure; HDL-c, high density lipoprotein cholesterol

Table S9: Mean trajectories of log fat mass and mean differences by maternal education, estimated from multilevel models

	Mean trajectory (95% CI) in less than O-level maternal education (reference)[a]	Mean difference in trajectory (95% CI) comparing with less than O-level maternal education[b]		
		O-level maternal education	A-level maternal education	Degree maternal education
**Females**				
Age 9yr (kg) or (%)	1.95 (1.91,1.99)	-3.00 (-7.60,1.60)	-4.99 (-9.78,-0.20)	-10.61 (-15.75,-5.48)
Change 9-13yr (kg/yr) or (%/yr)	0.18 (0.17,0.19)	-0.82 (-1.88,0.25)	-1.32 (-2.43,-0.21)	-0.79 (-2.03,0.46)
Change 13-15yr (kg/yr) or (%/yr)	0.14 (0.12,0.16)	0.94 (-1.08,2.95)	1.03 (-1.09,3.14)	1.88 (-0.47,4.23)
Change 15-18yr (kg/yr) or (%/yr)	0.08 (0.06,0.09)	-0.11 (-1.58,1.36)	-0.14 (-1.67,1.39)	-0.83 (-2.49,0.83)
Age 18yr (kg) or (%)	3.17 (3.13,3.22)	-4.68 (-9.57,0.21)	-8.43 (-13.42,-3.44)	-12.32 (-17.68,-6.96)
**Males**				
Age 9yr (kg) or (%)	1.54 (1.50,1.58)	2.09 (-2.77,6.94)	3.41 (-1.83,8.65)	-7.08 (-12.50,-1.66)
Change 9-13yr (kg/yr) or (%/yr)	0.19 (0.18,0.20)	-0.23 (-1.31,0.85)	-0.40 (-1.53,0.74)	-0.31 (-1.59,0.97)
Change 13-15yr (kg/yr) or (%/yr)	-0.02 (-0.04,0.00)	-0.69 (-2.74,1.36)	-1.87 (-3.97,0.23)	-0.16 (-2.54,2.22)
Change 15-18yr (kg/yr) or (%/yr)	0.09 (0.08,0.11)	-0.02 (-1.69,1.66)	0.63 (-1.08,2.33)	0.21 (-1.62,2.05)
Age 18yr (kg) or (%)	2.55 (2.50,2.60)	-0.28 (-5.91,5.35)	-0.14 (-6.01,5.72)	-7.94 (-13.97,-1.91)

CI, confidence interval; kg/yr, kilograms per year; %/yr, percentage per year.

[a]Fat mass was transformed using the natural log. All predicted mean values (kg) and rates of change per year (kg/yr) are on the log scale

[b]The difference between maternal education categories is back transformed from the log scale for ease of interpretation and is interpreted as the percentage difference in the mean level comparing each category with less than O-level maternal education or percentage difference in change per year (%/yr) comparing each category with maternal education.

Table S10: Mean trajectories of lean mass and mean differences by maternal education, estimated from multilevel models

	Mean trajectory (95% CI) in less than O-level maternal education (reference)	Mean difference in trajectory (95% CI) comparing with less than O-level maternal education		
		O-level maternal education	A-level maternal education	Degree maternal education
**Females**				
Age 9yr (kg)	23.37 (23.17,23.57)	-0.07 (-0.29,0.16)	-0.01 (-0.25,0.23)	0.03 (-0.24,0.29)
Change 9-13yr (kg/yr)	1.89 (1.79,1.98)	-0.04 (-0.14,0.06)	-0.03 (-0.14,0.07)	-0.01 (-0.13,0.11)
Change 13-15yr (kg/yr)	3.43 (3.26,3.60)	0.12 (-0.07,0.32)	0.09 (-0.12,0.29)	0.17 (-0.06,0.39)
Change 15-18yr (kg/yr)	0.09 (-0.04,0.21)	-0.01 (-0.16,0.15)	0.05 (-0.11,0.22)	0.07 (-0.11,0.24)
Age 18yr (kg)	38.02 (37.62,38.42)	0.01 (-0.50,0.52)	0.20 (-0.34,0.74)	0.51 (-0.09,1.11)
**Males**				
Age 9yr (kg)	30.49 (30.25,30.74)	0.18 (-0.04,0.40)	0.33 (0.09,0.57)	0.47 (0.19,0.74)
Change 9-13yr (kg/yr)	0.25 (0.15,0.34)	-0.02 (-0.12,0.08)	-0.05 (-0.16,0.06)	-0.15 (-0.27,-0.03)
Change 13-15yr (kg/yr)	5.49 (5.33,5.66)	0.12 (-0.08,0.32)	0.30 (0.09,0.51)	0.38 (0.15,0.61)
Change 15-18yr (kg/yr)	4.72 (4.56,4.87)	-0.04 (-0.22,0.13)	0.05 (-0.13,0.23)	-0.04 (-0.23,0.16)
Age 18yr (kg)	56.62 (56.16,57.07)	0.22 (-0.34,0.79)	0.87 (0.29,1.46)	0.52 (-0.13,1.17)

CI, confidence interval; kg/yr, kilograms per year

Table S11: Mean trajectories of SBP, DBP and pulse and mean differences by maternal education, estimated from multilevel models

	Mean trajectory (95% CI) in less than O-level maternal education (reference)[a]	Mean difference in trajectory (95% CI) comparing with less than O-level maternal education		
		O-level maternal education	A-level maternal education	Degree maternal education
**SBP**				
**Females**				
Age 7yr (mmHg)	99.29 (98.65,99.93)	-0.98 (-1.79,-0.16)	-1.71 (-2.57,-0.85)	-2.32 (-3.29,-1.34)
Change 7-12yr (mmHg/yr)	1.75 (1.60,1.91)	0.08 (-0.12,0.28)	0.13 (-0.08,0.34)	0.09 (-0.14,0.33)
Change 12-16yr (mmHg/yr)	3.76 (3.49,4.02)	0.13 (-0.20,0.46)	-0.03 (-0.38,0.31)	0.47 (0.08,0.85)
Change 16-18yr (mmHg/yr)	-5.14 (-5.71,-4.57)	-0.63 (-1.35,0.10)	-0.78 (-1.54,-0.01)	-1.53 (-2.38,-0.68)
Age 18yr (mmHg)	112.81 (112.03,113.59)	-1.30 (-2.29,-0.31)	-2.74 (-3.80,-1.69)	-3.04 (-4.21,-1.88)
**Males**				
Age 7yr (mmHg)	98.83 (98.20,99.45)	-0.64 (-1.43,0.16)	-1.30 (-2.15,-0.45)	-2.29 (-3.27,-1.31)
Change 7-12yr (mmHg/yr)	1.52 (1.36,1.67)	0.10 (-0.09,0.29)	0.23 (0.03,0.44)	0.24 (0.00,0.47)
Change 12-16yr (mmHg/yr)	5.59 (5.31,5.87)	0.21 (-0.14,0.56)	0.16 (-0.20,0.52)	0.30 (-0.11,0.70)
Change 16-18yr (mmHg/yr)	-3.26 (-3.92,-2.61)	-0.81 (-1.62,0.00)	-0.36 (-1.19,0.48)	-0.79 (-1.71,0.13)
Age 18yr (mmHg)	122.26 (121.33,123.18)	-0.94 (-2.09,0.21)	-0.21 (-1.39,0.98)	-1.50 (-2.80,-0.19)
**DBP**				
**Females**				
Age 7yr (mmHg)	57.78 (57.32,58.25)	-0.41 (-1.00,0.19)	-0.95 (-1.57,-0.32)	-1.36 (-2.07,-0.65)
Change 7-12yr (mmHg/yr)	0.07 (-0.05,0.19)	0.04 (-0.11,0.19)	0.08 (-0.08,0.24)	-0.03 (-0.21,0.15)
Change 12-16yr (mmHg/yr)	2.18 (1.95,2.40)	0.19 (-0.10,0.47)	-0.01 (-0.31,0.28)	0.51 (0.17,0.84)
Change 16-18yr (mmHg/yr)	-0.47 (-0.97,0.02)	-0.64 (-1.26,-0.02)	-0.60 (-1.26,0.06)	-1.28 (-2.01,-0.54)
Age 18yr (mmHg)	65.91 (65.34,66.47)	-0.76 (-1.48,-0.05)	-1.83 (-2.59,-1.06)	-2.05 (-2.90,-1.21)
**Males**				
Age 7yr (mmHg)	56.78 (56.32,57.24)	-0.41 (-0.99,0.17)	-0.69 (-1.31,-0.07)	-1.08 (-1.79,-0.37)
Change 7-12yr (mmHg/yr)	0.06 (-0.06,0.18)	0.08 (-0.07,0.23)	0.09 (-0.07,0.24)	0.02 (-0.16,0.20)
Change 12-16yr (mmHg/yr)	2.55 (2.31,2.80)	0.38 (0.07,0.68)	0.37 (0.06,0.69)	0.45 (0.10,0.81)
Change 16-18yr (mmHg/yr)	-1.75 (-2.30,-1.19)	-0.83 (-1.52,-0.14)	-0.96 (-1.68,-0.24)	-1.21 (-2.00,-0.41)
Age 18yr (mmHg)	63.81 (63.14,64.48)	-0.17 (-1.01,0.66)	-0.70 (-1.56,0.17)	-1.57 (-2.52,-0.62)
**Pulse rate**				
**Females**				
Age 7yr (bpm)	86.49 (85.73,87.24)	-0.74 (-1.70,0.23)	-0.89 (-1.90,0.13)	-0.52 (-1.68,0.63)
Change 7-12yr (bpm/yr)	-1.89 (-2.08,-1.71)	0.23 (0.00,0.47)	0.13 (-0.12,0.37)	0.00 (-0.27,0.28)
Change 12-16yr (bpm/yr)	-0.18 (-0.46,0.10)	-0.11 (-0.46,0.24)	-0.10 (-0.47,0.27)	0.04 (-0.37,0.45)
Change 16-18yr (bpm/yr)	-4.09 (-4.69,-3.48)	-0.37 (-1.13,0.39)	-1.05 (-1.86,-0.24)	-1.20 (-2.10,-0.30)
Age 18yr (bpm)	68.14 (67.28,69.00)	-0.76 (-1.85,0.33)	-2.74 (-3.91,-1.58)	-2.74 (-4.03,-1.45)
**Males**				
Age 7yr (bpm)	82.65 (81.91,83.39)	0.07 (-0.87,1.01)	0.01 (-0.99,1.02)	0.09 (-1.07,1.24)
Change 7-12yr (bpm/yr)	-1.94 (-2.12,-1.75)	-0.02 (-0.25,0.21)	-0.02 (-0.26,0.23)	0.10 (-0.18,0.38)
Change 12-16yr (bpm/yr)	-0.88 (-1.18,-0.58)	0.25 (-0.12,0.63)	0.17 (-0.22,0.55)	0.34 (-0.09,0.77)
Change 16-18yr (bpm/yr)	-3.54 (-4.23,-2.85)	-0.58 (-1.44,0.29)	-0.62 (-1.51,0.27)	-1.33 (-2.31,-0.35)
Age 18yr (bpm)	62.39 (61.37,63.41)	-0.16 (-1.43,1.12)	-0.64 (-1.96,0.67)	-0.73 (-2.17,0.71)

DBP, diastolic blood pressure; SBP, systolic blood pressure; CI, confidence interval

[a]Units are presented in mmHg for SBP and DBP and bpm for pulse rate

Table S12: Mean trajectories of glucose and mean differences by maternal education, estimated from multilevel models

	Mean trajectory (95% CI) in less than O-level maternal education (reference)	Mean difference in trajectory (95% CI) comparing with less than O-level maternal education		
		O-level maternal education	A-level maternal education	Degree maternal education
<u>**Glucose**</u>				
**Females**				
Age 7yr (mmol/l)	4.13 (4.09,4.17)	0.02 (-0.04,0.07)	-0.01 (-0.07,0.05)	-0.05 (-0.11,0.02)
Change 7-15yr (mmol/l/yr)	0.13 (0.12,0.14)	-0.004 (-0.01,0.01)	0.003 (-0.01,0.01)	0.01 (-0.003,0.02)
Change 15-18yr (mmol/l/yr)	-0.09 (-0.12,-0.07)	-0.001 (-0.03,0.03)	-0.01 (-0.04,0.02)	-0.01 (-0.05,0.02)
Age 18yr (mmol/l)	4.91 (4.86,4.96)	-0.01 (-0.07,0.05)	-0.02 (-0.08,0.05)	-0.01 (-0.08,0.06)
**Males**				
Age 7yr (mmol/l)	4.21 (4.17,4.25)	-0.03 (-0.08,0.02)	-0.004 (-0.06,0.05)	-0.04 (-0.10,0.03)
Change 7-15yr (mmol/l/yr)	0.14 (0.13,0.15)	0.004 (-0.01,0.01)	-0.0003 (-0.01,0.01)	0.003 (-0.01,0.02)
Change 15-18yr (mmol/l/yr)	-0.07 (-0.09,-0.04)	-0.02 (-0.05,0.02)	-0.004 (-0.04,0.03)	-0.02 (-0.06,0.01)
Age 18yr (mmol/l)	5.16 (5.10,5.21)	-0.05 (-0.11,0.02)	-0.02 (-0.08,0.05)	-0.09 (-0.16,-0.02)

CI, confidence interval; HDL-c, high density lipoprotein cholesterol; mmol/l, millimole per litre; %/yr, percentage per year

Table S13: Mean trajectories of HDL-c and non-HDL-c and mean differences by maternal education, estimated from multilevel models

	**Mean trajectory (95% CI) in less than O-level maternal education (reference)**	**Mean difference in trajectory (95% CI) comparing with less than O-level maternal education**		
		**O-level maternal education**	**A-level maternal education**	**Degree maternal education**
**HDL-c**				
**Females**				
Birth (mmol/l)	0.54 (0.52,0.56)	-0.02 (-0.04,0.01)	0.02 (-0.01,0.05)	0.03 (-0.001,0.06)
Change 0-7yr (mmol/l/yr)	0.13 (0.13,0.14)	0.004 (-0.001,0.01)	-0.004 (-0.01,0.002)	-0.004 (-0.01,0.003)
Change 7-18yr (mmol/l/yr)	-0.02 (-0.02,-0.01)	0.002 (-0.002,0.01)	0.01 (0.002,0.01)	0.01 (0.01,0.01)
Age 18yr (mmol/l)	1.28 (1.25,1.30)	0.03 (0.001,0.06)	0.05 (0.02,0.08)	0.11 (0.07,0.15)
**Males**				
Birth (mmol/l)	0.49 (0.47,0.51)	0.02 (0.0003,0.05)	0.02 (-0.002,0.05)	0.01 (-0.02,0.04)
Change 0-7yr (mmol/l/yr)	0.15 (0.15,0.16)	-0.005 (-0.01,-0.0003)	-0.01 (-0.01,-0.0003)	-0.01 (-0.01,0.001)
Change 7-18yr (mmol/l/yr)	-0.04 (-0.04,-0.03)	0.001 (-0.003,0.004)	0.002 (-0.002,0.01)	0.003 (-0.001,0.01)
Age 18yr (mmol/l)	1.15 (1.12,1.18)	-0.01 (-0.04,0.03)	0.002 (-0.03,0.04)	0.005 (-0.03,0.04)
**Non-HDL-c**				
**Females**				
Birth (mmol/l)	1.28 (1.23,1.32)	0.01 (-0.05,0.06)	0.06 (-0.01,0.12)	0.02 (-0.05,0.10)
Change 0-9yr (mmol/l/yr)	0.21 (0.20,0.22)	-0.003 (-0.01,0.01)	-0.01 (-0.02,0.003)	-0.01 (-0.03,-0.002)
Change 9-18yr (mmol/l/yr)	-0.07 (-0.08,-0.06)	-0.006 (-0.01,0.01)	-0.01 (-0.01,0.004)	0.002 (-0.01,0.01)
Age 18yr (mmol/l)	2.55 (2.49,2.61)	-0.05 (-0.12,0.02)	-0.09 (-0.16,-0.01)	-0.09 (-0.18,-0.01)
**Males**				
Birth (mmol/l)	1.22 (1.18,1.26)	0.002 (-0.05,0.06)	0.02 (-0.05,0.08)	0.01 (-0.07,0.09)
Change 0-9yr (mmol/l/yr)	0.19 (0.19,0.20)	-0.002 (-0.01,0.01)	-0.005 (-0.01,0.01)	-0.003 (-0.01,0.01)
Change 9-18yr (mmol/l/yr)	-0.07 (-0.08,-0.06)	-0.01 (-0.01,0.004)	-0.002 (-0.01,0.01)	0.001 (-0.01,0.01)
Age 18yr (mmol/l)	2.32 (2.26,2.38)	-0.06 (-0.13,0.02)	-0.04 (-0.13,0.03)	-0.01 (-0.9,0.08)

CI, confidence interval; HDL-c, high density lipoprotein cholesterol; mmol/l, millimole per litre

Table S14: Mean trajectories of triglycerides by maternal education, estimated from multilevel models

	Mean trajectory (95% CI) in less than O-level maternal education (reference)[a]	Mean difference in trajectory (95% CI) comparing with less than O-level maternal education [b]		
		O-level maternal education	A-level maternal education	Degree maternal education
**Female**				
Birth (mmol/l or %)	-0.70 (-0.73,-0.66)	2.40 (-2.43,7.22)	4.68 (-0.93,10.28)	0.31 (-5.98,6.60)
Change 0-9yr (mmol/l/yr or %/yr)	0.09 (0.08,0.10)	-0.45 (-1.17,0.27)	-0.56 (-1.36,0.24)	-0.23 (-1.16,0.69)
Change 9-18yr (mmol/l/yr or %/yr)	-0.04 (-0.05,-0.04)	0.09 (-0.58,0.75)	-0.33 (-1.02,0.36)	-0.62 (-1.37,0.14)
Age 18yr (mmol/l or %)	-0.26 (-0.30,-0.23)	-0.91 (-5.36,3.54)	-3.37 (-7.91,1.17)	-7.11 (-11.86,-2.35)
**Male**				
Birth (mmol/l or %)	-0.68 (-0.72,-0.65)	1.31 (-3.37,6.00)	0.14 (-5.03,5.31)	3.53 (-3.19,10.26)
Change 0-9yr (mmol/l/yr or %/yr)	0.08 (0.07,0.08)	-0.10 (-0.81,0.61)	0.32 (-0.45,1.10)	0.04 (-0.90,0.98)
Change 9-18yr (mmol/l/yr or %/yr)	-0.04 (-0.04,-0.03)	-0.23 (-0.91,0.45)	-0.52 (-1.21,0.18)	-0.31 (-1.08,0.46)
Age 18yr (mmol/l or %)	-0.32 (-0.36,-0.29)	-1.66 (-6.40,3.08)	-1.59 (-6.37,3.19)	1.07 (-4.30,6.45)

CI, confidence interval; mmol/l, millimole per litre; mmol/l/year; %/yr, percentage per year

[a]Triglyceride was transformed using the natural log. All predicted mean values (mmol/l) and rates of change per year (mmol/l/yr) are on the log scale

[b]The difference by maternal education is back transformed from the log scale for ease of interpretation and is interpreted as the percentage difference in the mean level comparing each category with less than O-level maternal education or percentage difference in change per year (%/yr) comparing each category with less than O-level maternal education

Table S15: Characteristics at birth of the mothers of children included in models compared with those excluded due to missing exposure or outcome data

	Included[a]	Excluded[a]
	**n (%)**	**n (%)**
**Maternal marital status**		
Never married	1348(15.4)	1222(25.9)
Separated/Divorced/Widowed	453(5.2)	356(7.5)
1st Marriage	6368(72.8)	2844(60.2)
Marriage 2 or 3	576(6.6)	302(6.4)
**Household social class †**		
Professional	1237(14.7)	297(9.5)
Managerial & Technical	3632(43.2)	1179(37.8)
Non-Manual	2110(25.1)	832(26.6)
Manual	1008(12.0)	552(17.7)
Part Skilled & Unskilled	430(5.1)	262(8.4)
**Maternal education**		
Less than O level	2309(25.8)	1434(41.1)
O level	3139(35.1)	1172(33.6)
A level	2198(24.6)	589(16.9)
Degree or above	1306(14.5)	298(8.5)
**Mother's partner's education**		
Less than O level	2675(31.0)	1464(44.0)
O level	1866(21.6)	682(20.5)
A level	2349(27.2)	759(22.8)
Degree or Above	1746(20.2)	425(12.8)
**Maternal smoking during pregnancy**		
No	6965(79.3)	2972(66.2)
Yes	1891(20.7)	1519(33.8)
**Parity**		
0	3859(43.8)	1975(30.1)
1	3148(35.7)	1416(21.6)
2+	1799(20.4)	3175(48.4)
**Breastfeeding**		
Exclusive	2540(31.9)	667(22.3)
Non-exclusive	3866(48.5)	1454(46.2)
Never	1562(19.6)	980(31.3)
	**Mean (SD)**	**Mean (SD)**
**Gestational age (weeks)**	39(1.8)	37(8.3)
**Birth weight (g)**	3429(535.2)	3293(643.8)
**Maternal pre-pregnancy BMI (kg/m^2)**	23(3.5)	23(3.6)
**Maternal age at delivery (years)**	29(4.7)	27(5.2)

[a] Denominators for excluded participants in this table vary due to different rates of missing data for characteristics shown. Number of participants included in analyses of triglycerides (n=8,952) used as the denominator in this table given the varying sample sizes included in analyses.

† Household social class was measured as the highest of the mother's or her partner's occupational social class using data on job title and details of occupation. Social class was derived using the standard occupational classification (SOC) codes developed by the United Kingdom Office of Population Census and Surveys and classified as I professional, II managerial and technical, IIINM non-manual, IIIM manual, and IV&V part skilled occupations and unskilled occupations.
